# Growth‐Pathway‐Controlled van der Waals Epitaxy of Phase‐Selective Tin Sulfides

**DOI:** 10.1002/advs.76199

**Published:** 2026-06-26

**Authors:** Jaehyeok Lee, Jinwoo Kim, Gwan‐Hyoung Lee

**Affiliations:** ^1^ Department of Material Science and Engineering Seoul National University Seoul Republic of Korea

**Keywords:** growth pathway, heterostructures, phase engineering, tin sulfide, van der Waals epitaxy

## Abstract

Controlling crystalline phase and interfacial strain remains a central challenge in van der Waals (vdW) epitaxy for polymorphic two‐dimensional (2D) materials. Tin sulfides represent an ideal platform, yet deterministic phase control and growth‐pathway effects on phase stability and strain accommodation remain unexplored. Here, we demonstrate growth‐pathway‐controlled vdW epitaxy of phase‐selective tin sulfides on WSe_2_ using a two‐zone chemical vapor deposition (CVD) system. Under sulfur‐rich conditions, tin sulfide adopts a hexagonal SnS_2_ structure that shares the trigonal symmetry of WSe_2_, enabling a unique in‐plane epitaxial alignment despite a large lattice mismatch. Under sulfur‐deficient conditions, tin sulfide stabilizes in orthorhombic SnS, whose symmetry is incompatible with WSe_2_. As a result, SnS nucleates with multiple energetically comparable epitaxial registries. Direct growth of SnS on WSe_2_ generates substrate‐mediated strain, leading to local lattice distortion and phase evolution from α‐ to β′‐SnS. Through sequential control of the precursor temperature, SnS is grown on a pre‐formed SnS_2_ rather than directly on WSe_2_. This sequential growth suppresses substrate‐induced strain and yields deformation‐free α‐SnS via a SnS_2_ vdW buffer layer. These findings establish growth‐pathway‐controlled vdW epitaxy as a strategy for decoupling epitaxial alignment from strain accommodation, enabling deterministic phase control and structural integrity in lattice‐mismatched vdW heterostructures.

## Graphical abstract

Growth‐pathway‐controlled van der Waals epitaxy enables deterministic phase selection and strain engineering in tin sulfide/WSe_2_ heterostructures. Direct growth of SnS induces substrate‐mediated strain and phase evolution, whereas sequential growth through an SnS_2_ buffer suppresses strain transfer and stabilizes pristine α‐SnS, revealing a versatile route to structurally controlled lattice‐mismatched heterostructures.

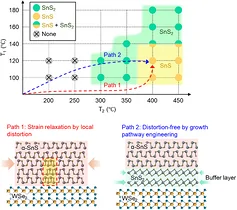

## 1 Introduction

Van der Waals (vdW) epitaxy of two‐dimensional (2D) materials provides a powerful platform for constructing lattice‐mismatched heterostructures with atomically sharp interfaces, enabling access to interfacial phenomena beyond the constraints of conventional epitaxy [1, 2, 3, 4, 5]. Although vdW interactions relax the requirement for strict lattice matching, interfacial strain can still arise from lattice and symmetry mismatch at vdW interfaces, influencing structural stability and phase evolution in overgrown 2D layers [6, 7, 8, 9]. Among various vdW materials, tin sulfides (SnS and SnS_2_) offer a vdW epitaxial system where multiple structural phases can be selectively synthesized [10, 11]. This phase tunability makes tin sulfides a suitable platform for investigating phase formation and structural stability under vdW epitaxy. In addition to this epitaxial versatility, SnS and SnS_2_ exhibit complementary p‐n characteristics and strong optical absorption [10, 12, 13, 14]: orthorhombic SnS is p‐type semiconductor with pronounced in‐plane anisotropy and ferroelectricity [15, 16, 17], whereas hexagonal 1T‐SnS_2_ is an n‐type semiconductor [14, 18]. While previous studies have demonstrated aligned vdW epitaxy and transformation‐driven orientational selection in SnS/SnS_2_ systems [12, 19, 20], the role of growth pathways in governing phase stability and interfacial strain accommodation during vdW epitaxy remains largely unexplored. In particular, deterministic control of strain propagation and phase evolution through pathway engineering during chemical vapor deposition has not yet been systematically established.

Here, we demonstrate growth‐pathway‐controlled vdW epitaxy of phase‐selective tin sulfides on WSe_2_ using a two‐zone chemical vapor deposition (CVD) system with independent control of sulfur and tin precursor supply at different zone temperatures. By tuning the precursor supply, we achieve selective formation of SnS or SnS_2_ on WSe_2_ despite their large lattice mismatch. More importantly, controlling the growth pathway by independently and sequentially modulating the sulfur and tin precursor supply allows SnS to follow two distinct growth scenarios: direct nucleation of SnS on the WSe_2_ substrate or overgrowth of SnS on a pre‐formed SnS_2_ layers, leading to SnS/SnS_2_/WSe_2_ heterostructure. These two pathways produce fundamentally different interfacial structures. When SnS nucleates directly on WSe_2_, interfacial structural incompatibility gives rise to lattice distortion and a strain‐driven phase evolution within the SnS layer. In contrast, when SnS grows on an intermediate SnS_2_ layers, the SnS_2_ effectively decouples SnS from the WSe_2_ substrate, suppressing strain transfer and stabilizing distortion‐free α‐SnS in a vertically stacked SnS/SnS_2_/WSe_2_ heterostructure.

## 2 Results and Discussion

**FIGURE 1:**
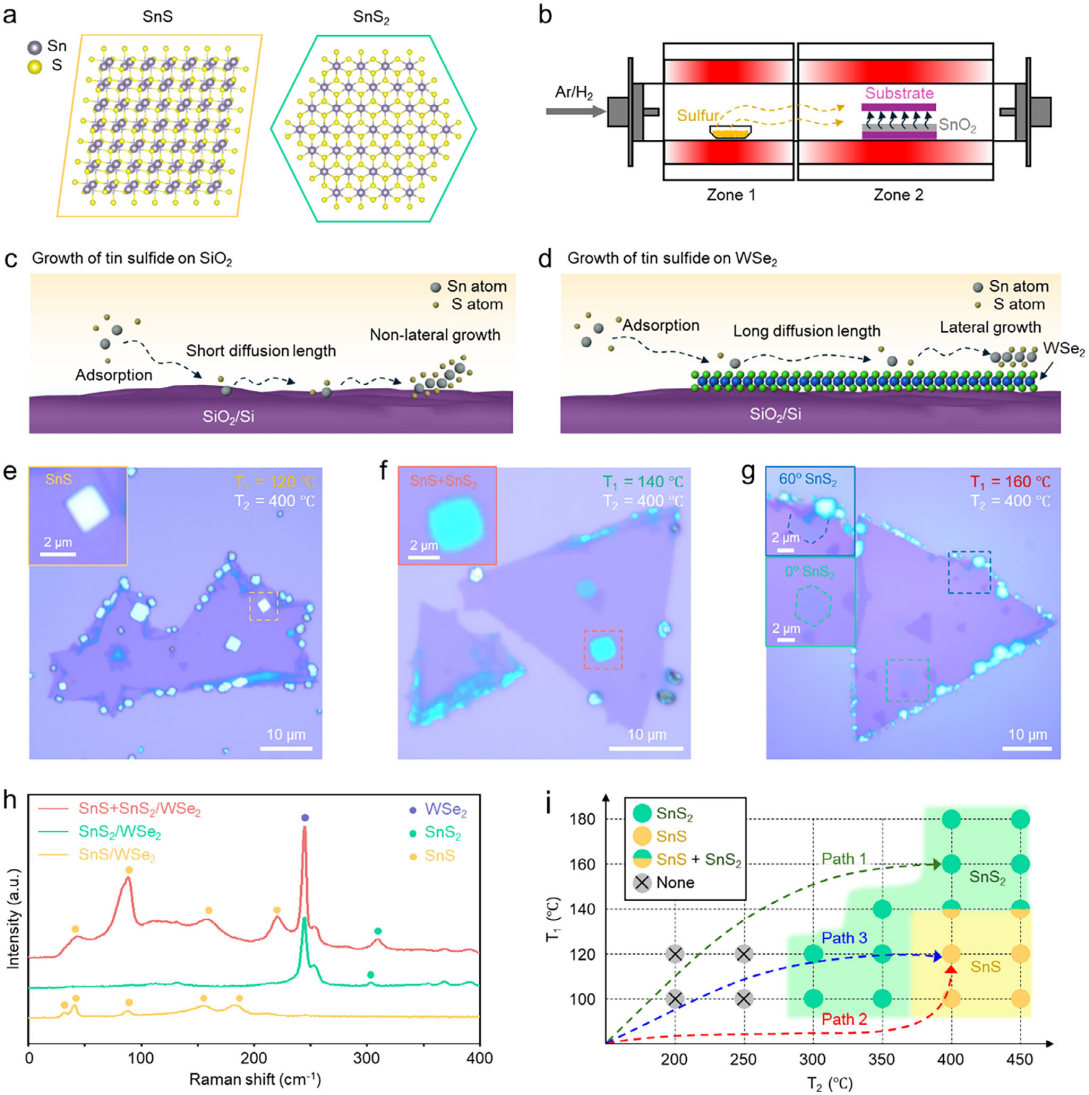
Growth‐pathway‐controlled van der Waals epitaxy of tin sulfides and optimized conditions. (a) Atomic illustration of SnS and SnS_2_ with their S zig‐zag edge. (b) Schematic of two‐zone CVD system for growth of tin sulfide. The temperature of each zone is controlled independently. (c, d) Schematic illustration of (c) non‐lateral growth of tin sulfide on a SiO_2_ substrate and (d) van der Waals epitaxy of tin sulfide on a 2D substrate. (e–g) Optical microscopy images of (e) SnS grown on CVD‐grown WSe_2_ at T_1_ = 120°C and T_2_ = 400°C, (f) SnS+SnS_2_ grown on WSe_2_ at T_1_ = 140°C and T_2_ = 400°C, (g) SnS_2_ grown on WSe_2_ at T_1_ = 160°C and T_2_ = 400°C. Each inset shows a magnified optical image of the heterostructure. (h) Raman spectra of SnS on WSe_2_, SnS_2_ on WSe_2_, and SnS+SnS_2_ on WSe_2_. (i) Temperature‐dependent phase map and growth pathways of SnS, SnS_2_, and SnS/SnS_2_.

Two representative crystal structures of tin sulfides investigated in this study are illustrated in Figure 1a: orthorhombic SnS and hexagonal SnS_2_. Although both phases terminate with sulfur zigzag edges, their distinct crystal symmetries lead to different equilibrium morphologies. Previous studies have shown that orthorhombic SnS typically forms rhombic flakes [15, 16, 17, 19, 21, 22, 23, 24, 25], whereas hexagonal 1T‐SnS_2_ preferentially exhibits triangular or hexagonal shapes [12, 14, 26, 27].

The fundamental difference between these two crystal structures originates from their distinct chemical stoichiometries, which are governed by the atomic ratio (Sn:S) [11, 12, 19]. To selectively control the phase of tin sulfide, a two‐zone chemical vapor deposition (CVD) system was employed to independently regulate the sulfur and tin precursor supply (Figure 1b). As the vapor pressure of sulfur is highly dependent on temperature, precise control of T_1_ is essential to achieve the targeted stoichiometry of tin sulfides. In this configuration, sulfur powder was placed in the upstream zone (Zone 1), while a 10 nm‐thick SnO_2_ thin film deposited on a SiO_2_ substrate served as the tin source in the downstream zone (Zone 2). The substrate was positioned face‐down above the SnO_2_ source using a sapphire spacer, and a mixed Ar/H_2_ carrier gas was introduced during growth. This dual‐heating setup allows for the precise and independent optimization of both precursor flux and the substrate temperature, providing the technical basis for the deterministic phase engineering.

To achieve high‐quality growth of tin sulfides, the selection of the growth substrate is critical for facilitating vdW epitaxy, as schematically illustrated in Figure 1c,d. Under identical growth conditions, tin sulfide flakes grown on amorphous SiO_2_ exhibit high nucleation density with random in‐plane orientations, whereas those grown on crystalline WSe_2_ show laterally extended, vdW‐epitaxially aligned crystals. This distinct growth mode is experimentally confirmed by comparing the results on SiO_2_ and various 2D substrates (Figure S1), highlighting the role of the growth substrate in determining nucleation density and alignment. This difference is attributed to the distinct surface characteristics; SiO_2_ contains a high density of surface dangling bonds that act as trapping sites, thereby limiting surface diffusion and resulting in random crystalline orientations. In contrast, atomically flat 2D substrates like WSe_2_ provide chemically inert surfaces free of dangling bonds, enabling long diffusion lengths and selective nucleation at energetically favorable sites, particularly along the step edges. The periodic potential of the crystalline 2D layers further promotes vdW epitaxial alignment rather than randomly oriented growth [28].

Utilizing the CVD‐grown WSe_2_ as a growth template for vdW epitaxy within the two‐zone CVD system, we examined the growth outcomes at a fixed substrate temperature (T_2_ = 400°C) while varying the sulfur‐source temperature (T_1_) as presented in Figure 1e–g. At T_1_ = 120°C, the flakes exhibit predominantly rhombic morphologies, characteristic of orthorhombic SnS (Inset of Figure 1e). In contrast, at T_1_ = 160°C, triangular and hexagonal flakes are dominantly observed, indicating the formation of hexagonal SnS_2_ (Inset of Figure 1g). At an intermediate temperature of T_1_ = 140°C, both morphologies coexist within individual flakes, suggesting that the growth condition lies near a phase boundary (Insets of Figure 1f).

To confirm the correlation between morphologies and crystal phases, Raman spectroscopy was performed (Figure 1h). The sample grown at T_1_ = 120°C exhibits the characteristic A_g_ (∼41, ∼88, ∼185 cm^−1^) and B_3g_ (∼155 cm^−1^) modes of orthorhombic SnS, while the sample grown at T_1_ = 160°C shows the distinct A_1g_ mode (∼310 cm^−1^) of hexagonal SnS_2_. Notably, both sets of Raman modes are detected at the same spatial location for the T_1_ = 140°C sample, confirming the coexistence of SnS and SnS_2_ within a single flake. These results establish that the observed morphology differences directly reflect the phase selectivity controlled by the sulfur supply.

By integrating these systematic observations, a comprehensive phase map for tin sulfide growth was constructed (Figure 1i). The phase map is categorized into three distinct regimes based on the growth outcomes: None (no growth of tin sulfide as marked with ‘ × ’), SnS_2_ (green circles), and SnS (orange circles), with a phase boundary between the two phases. When T_1_< 80°C or T_2_< 250°C, no tin sulfide forms, indicating insufficient precursor supply for nucleation. Once T_2_ exceeds ∼250°C, SnS_2_ becomes the thermodynamically favored phase over a broad range of T_1_ values in the intermediate temperature regime (250°C < T_2_ ≤ 350°C), suggesting that under these conditions the sulfur chemical potential remains sufficiently high to stabilize the sulfur‐rich SnS_2_ phase. As T_2_ increases further (T_2_ ≥ 400°C), the growth outcome becomes increasingly sensitive to T_1_, which controls the sulfur vapor pressure. In this high‐temperature regime, three subregions emerge: At low T_1_ (sulfur‐deficient conditions), orthorhombic SnS is stabilized as the dominant phase. At high T_1_ (sulfur‐rich conditions), hexagonal SnS_2_ remains stable. At intermediate T1 (∼140°C), a mixed‐phase region appears, where SnS, SnS_2_, and a mixture of SnS and SnS_2_ coexist within individual flakes, defining a finite phase boundary region rather than a sharp transition line. This phase boundary reflects the competition between sulfur supply (governed by T_1_) and tin precursor activation (governed by T_2_), which together determine the relative stability of the SnS and SnS_2_ polymorphs. Mapping this interdependent parameter space provides a strategic framework for designing growth pathways that selectively navigate through single‐phase or mixed‐phase regions to achieve deterministic phase control.

Based on this map, we designed three distinct growth pathways (Paths 1–3) to navigate the T_1_‐T_2_ parameter space for deterministic phase selection. Path 1 is designed to synthesize phase‐pure SnS_2_ by maintaining a high sulfur supply throughout the heating process, ensuring the growth remains consistently within the SnS_2_ stability window. In contrast, Path 2 is engineered to selectively grow phase‐pure SnS by delaying the temperature rise of T_1_ to limit the sulfur supply while T_2_ increases to the high‐temperature regime. This heating trajectory is specifically intended to bypass the SnS_2_ regime during the ramp‐up. Path 3 is designed to first form SnS_2_ on WSe_2_ and subsequently grow SnS by deliberately shifting the growth trajectory across the phase boundary from the SnS_2_ regime to the SnS regime during temperature ramping, thereby yielding vertically stacked SnS/SnS_2_ heterostructures. By governing the heating trajectory through these designed pathways, we can achieve the deterministic phase‐controlled vdW epitaxy of the desired tin sulfide phases.

**FIGURE 2:**
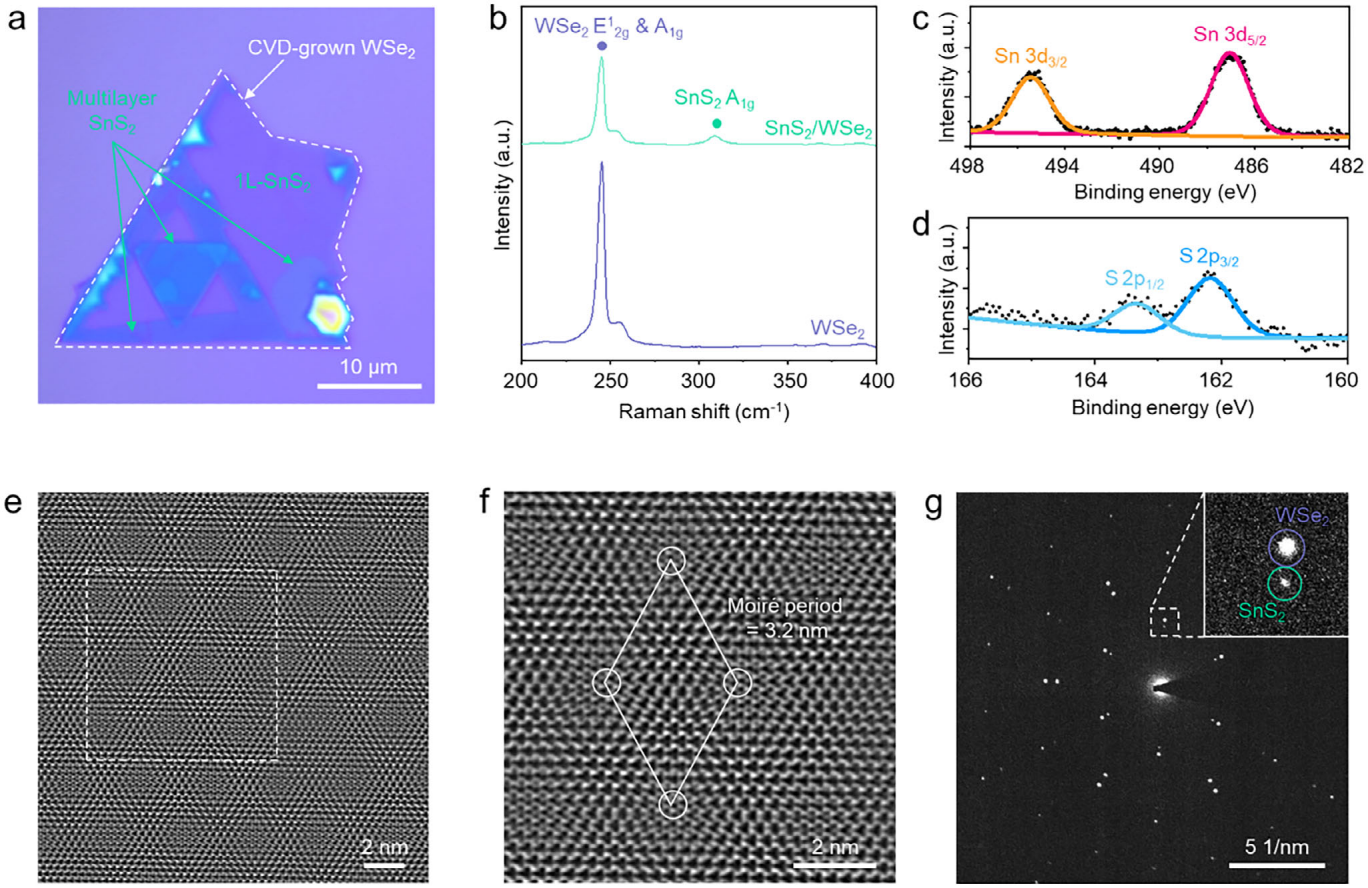
Epitaxial growth of SnS_2_ on WSe_2_. (a) OM image of monolayer (1L) and multilayer SnS_2_ on WSe_2._ (b) Raman spectrum of 1L and multilayer SnS_2_ on WSe_2_. (c, d) XPS of SnS/WSe_2_, showing spectrum of (c) Sn 3d and (d) S 2p, respectively. (e) Low‐magnification HAADF‐STEM image of SnS_2_/WSe_2_ heterostructure revealing uniform moiré pattern. (f) Magnified image of boxed region in (f), which reveals a periodic moiré pattern with approximately 3.2 nm period. (g) Selected‐area electron diffraction (SAED) pattern of SnS_2_/WSe_2_ exhibiting 0° epitaxial alignment with inset showing magnified view of first diffraction point of WSe_2_ and SnS_2_.

To examine the epitaxial characteristics of SnS_2_ grown on WSe_2_, we synthesized SnS_2_ on CVD‐grown WSe_2_ under sulfur‐rich conditions following Path 1 (T_1_ = 160°C and T_2_ = 400°C). As shown in Figure 2a, the WSe_2_ is fully covered by monolayer SnS_2_ (1L‐SnS_2_) with a few multilayer patches (multilayer‐SnS_2_), which have a step‐like thickness of 0.6 nm as confirmed by atomic force microscopy (AFM) in Figure S2. Raman spectra in Figure 2b confirm the formation of SnS_2_/WSe_2_ heterostructure, showing the characteristic A_1g_ mode of SnS_2_ [18, 26, 27]. To evaluate the chemical state and stoichiometry, x‐ray photoelectron spectroscopy (XPS) was performed (Figure 2c,d). The Sn 3d and S 2p peaks corresponding to the Sn^4+^ and S^2−^ states are observed, confirming the formation of SnS_2_ on WSe_2_.

To further investigate the crystallographic relationship between SnS_2_ and the WSe_2_, we used scanning transmission electron microscopy (STEM) as shown in Figure 2e–g. High‐angle annular dark‐field (HAADF) STEM image of Figure 2e shows a spatially uniform moiré pattern over a large area, indicating the absence of significant local deformation in the SnS_2_/WSe_2_ heterostructure. Figure 2f presents a magnified image of the white dashed box in Figure 2e, revealing a moiré period of approximately 3.2 nm. Selected‐area electron diffraction (SAED) patterns (Figure 2g) show that the SnS_2_ and WSe_2_ are aligned with zero twist angle despite the substantial lattice mismatch of 11%, which is calculated from lattice constant of 0.329 nm for WSe_2_ and 0.366 nm for SnS_2_ in SAED patterns. The moiré period (*L*) of zero‐twist SnS_2_/WSe_2_ heterobilayer, 3.2 nm, directly measured in STEM image agrees well with the moiré periodicity calculated from the equation, $L=\frac{a_{WSe_{2}}\times a_{SnS_{2}}}{a_{SnS_{2}}-a_{WSe_{2}}}$ (Figure 2f) [29]. The indicates that the SnS_2_ and WSe_2_ layers remain nearly strain‐free, which is the characteristics of van der Waals epitaxy.

**FIGURE 3:**
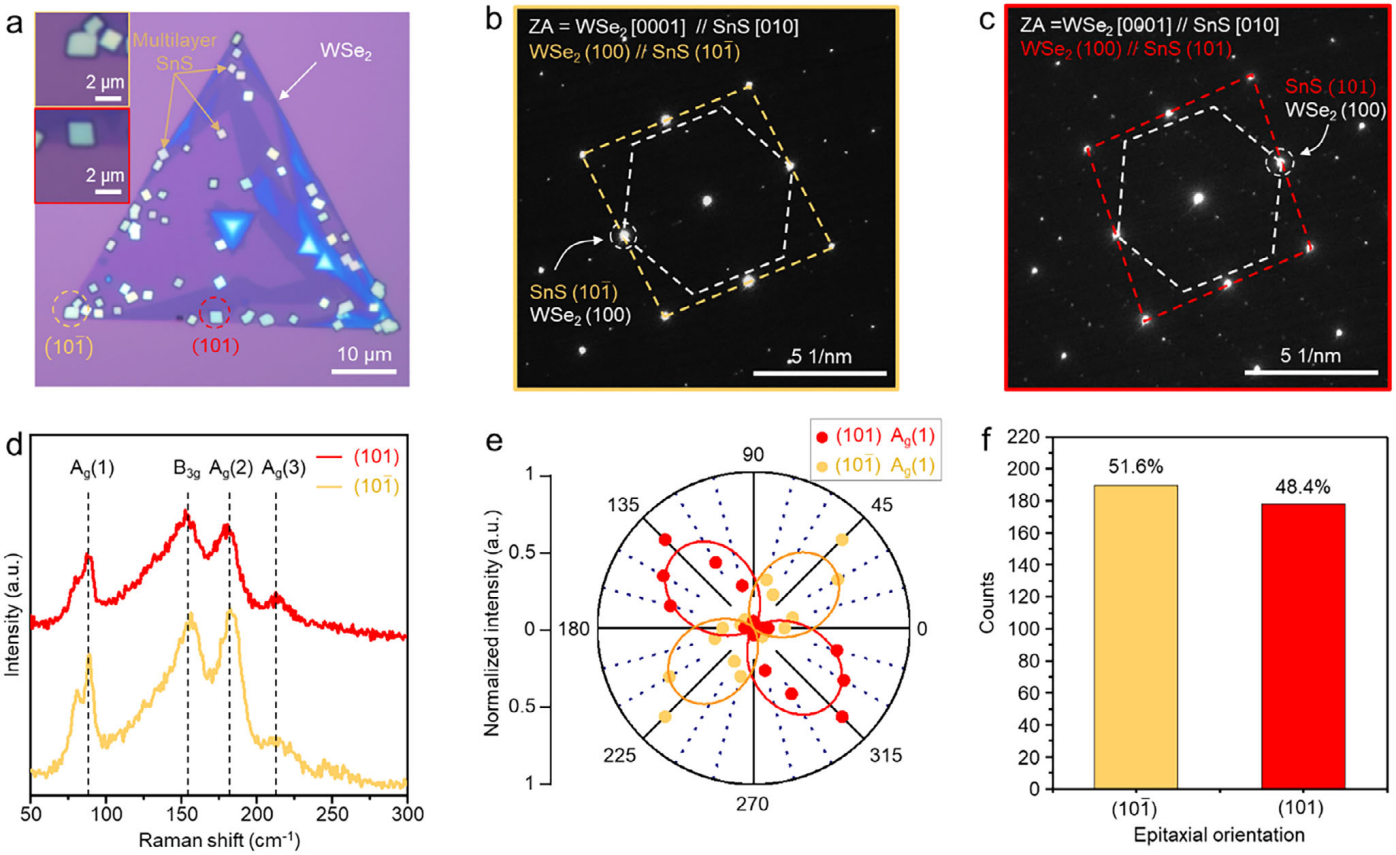
Epitaxial growth of SnS on WSe_2_. (a) OM image of SnS grown on WSe_2_. The yellow and red dashed circles indicate SnS flakes with the $\left(10\bar{1}\right)$ and (101) planes aligned to the (100) plane of WSe_2_, respectively. The insets are magnified views of different SnS aligned with WSe_2_. (b, c) SAED pattern of SnS/WSe_2_, where (b) SnS $\left(10\bar{1}\right)$ plane is aligned with the WSe_2_ (100) direction, (c) SnS (101) plane is aligned with the WSe_2_ (100) direction. (d) Raman spectra of the SnS domains marked by the yellow and red dashed circles in (a). (e) Polar plot of angle‐dependent polarized Raman spectra of the two SnS domains, showing the A_g_(1) mode with 2‐fold symmetry along the armchair direction. (f) Bar chart showing the distribution of the two representative in‐plane SnS orientations aligned on the (100) plane of WSe_2_.

When orthorhombic SnS was synthesized on WSe_2_ under sulfur‐deficient conditions by Path 2, SnS forms rhombic crystals that exhibit multiple in‐plane orientations on the same WSe_2_ substrate, as shown in Figure 3a. This orientation multiplicity reflects the symmetry mismatch between two‐fold symmetric orthorhombic SnS and the three‐fold symmetric WSe_2_ surface. To directly resolve the atomic structure of the grown SnS flakes and the epitaxial relationship between SnS and WSe_2_, HAADF‐STEM images were obtained (Figure S3). SAED patterns of two representative SnS flakes in Figure 3b,c show the epitaxial relationship between SnS and WSe_2_: The $\left(10\bar{1}\right)$ and (101) planes of SnS are aligned with the (100) plane of WSe_2_ in yellow and red dashed flakes of Figure 3a, respectively. The insets in Figure 3a show magnified views of the red and yellow dashed flakes that share the trigonal symmetry underlying WSe_2_ crystallographic plane, but adopt distinct in‐plane orientations.

Raman spectroscopy was employed to verify the crystal phase and anisotropy of the SnS crystals with different in‐plane orientations. As shown in Figure 3d, the Raman spectra obtained from the individual flakes highlighted by red and yellow dashed circles in Figure 3a exhibit the characteristic A_g_(1), B_3g_, A_g_(2), A_g_(3) vibrational modes of orthorhombic SnS [22, 23, 30, 31]. To further resolve their in‐plane crystallographic orientation on the WSe_2_, angle‐resolved polarized Raman (ARPR) spectroscopy was used, as shown in the ARPR spectra (Figure S4), and the polar plots of the A_g_(1) mode of each SnS flake (Figure 3e). All measurements were conducted under a parallel polarization configuration with in‐plane rotation of the sample to investigate the anisotropy of SnS. Both crystals yellow and red dashed circles, denoted as $\left(10\bar{1}\right)$ and (101), respectively, exhibit a pronounced two‐fold symmetry, consistent with the armchair‐direction‐dependent Raman response of the orthorhombic structure of SnS [23]. However, the directions of maximum Raman intensity are rotated by ∼90° relative to each other, indicating that the two SnS crystals adopt distinct in‐plane orientations while maintaining epitaxial alignment along *z*‐axis on the WSe_2_. While the two‐fold symmetry of the A_g_(1) mode clearly reflects this 90° rotation, the B_3g_ mode remains nearly unchanged due to its inherent four‐fold symmetry (Figure S5).

To establish the statistical generality of the two epitaxial registries identified above, the in‐plane crystallographic orientations of SnS flakes grown on WSe_2_ were systematically analyzed. The flakes are categorized into two primary epitaxial registries based on their alignment with the substrate. Statistical analysis of 368 SnS crystals reveals that 190 flakes (51.6%) adopt the $\left(10\bar{1}\right)$//(100) alignment, while 178 flakes (48.4%) follow the (101)//(100) alignment (Figure 3f). These results indicate that the two epitaxial orientations occur with nearly equal probability. This orientational degeneracy directly arises from the symmetry mismatch between the two‐fold symmetric orthorhombic SnS and the three‐fold symmetric WSe_2_ surface. Such findings demonstrate that SnS can accommodate two energetically comparable epitaxial alignments on a three‐fold symmetric template, highlighting how substrate symmetry governs registry selection even at the weakly bonded van der Waals interface. These results demonstrate that two‐fold symmetric SnS can accommodate two energetically comparable epitaxial alignments on a three‐fold symmetric WSe_2_ substrate, highlighting the role of symmetry mismatch in van der Waals epitaxy.

**FIGURE 4:**
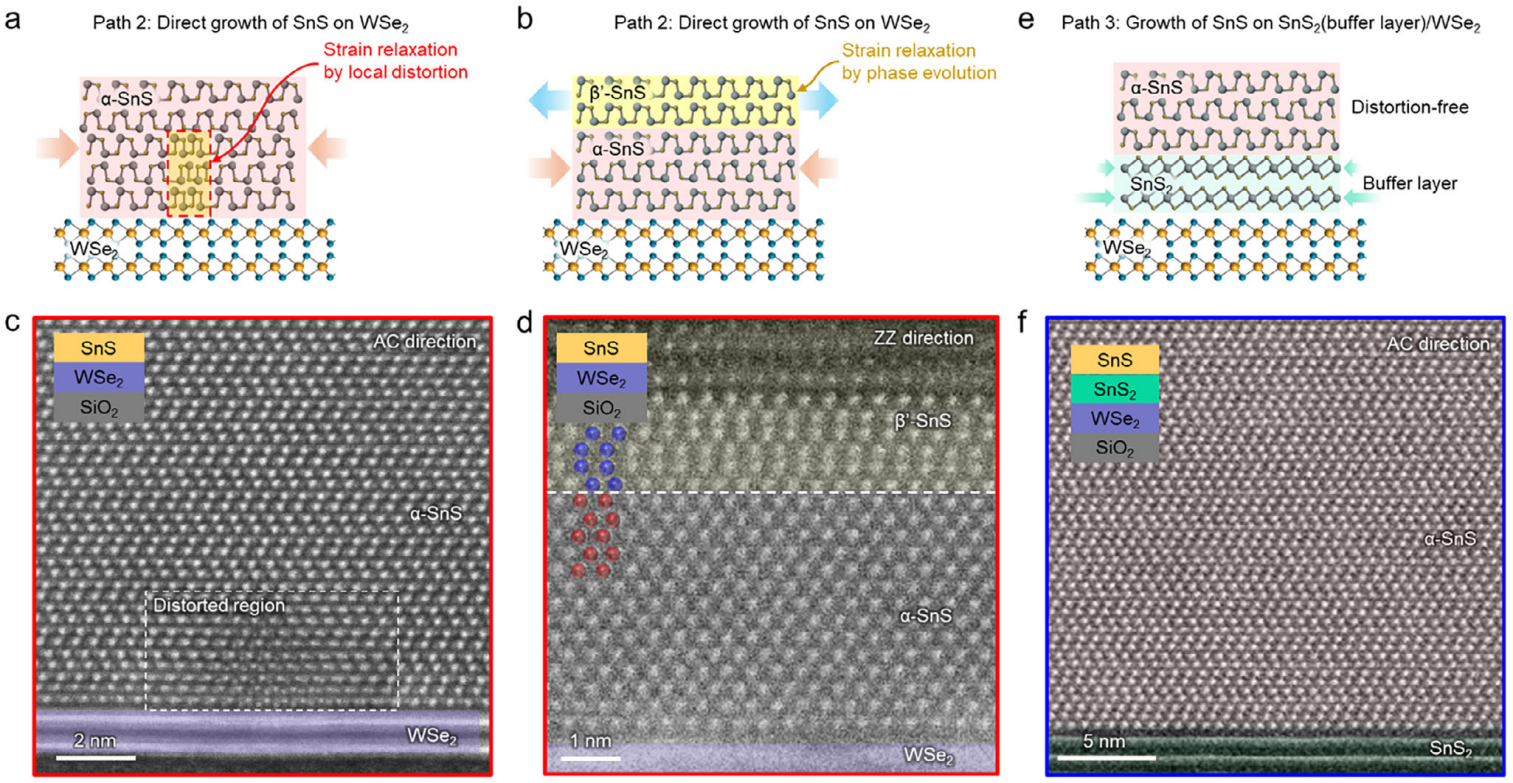
Pathway controlled growth heterostructure and resulting atomic structures of SnS on WSe_2_. (a‐b) Schematic illustrations of growth behavior on Path 2: Direct nucleation on WSe_2_ where lattice‐mismatch induced strain leads to structural relaxation through (a) the formation of interfacial dislocations or (b) phase evolution. (c, d) Cross‐sectional HAADF‐STEM images confirming the structural characteristics of Path 2: (c) α‐SnS layer showing a localized distorted region along the armchair (AC) direction due to substrate‐induced strain. (d) Interface between β'‐SnS and α‐SnS observed along the zigzag (ZZ) direction, corresponding to the strain relaxation via stacking variations. Sn atoms are highlighted with red and blue circles. (e) Schematic illustrations of growth behavior on Path 3: Buffer‐mediated growth utilizing a SnS_2_ vdW layer, which facilitates the synthesis of distortion‐free SnS by decoupling the lattice interaction with the substrate. (e) Distortion‐free α‐SnS grown on a SnS_2_ buffer layer along the AC direction, demonstrating the effectiveness of the vdW buffer strategy. (f) Cross‐sectional HAADF‐STEM images confirming the structural characteristics of Path 3.

To clarify how interfacial strain governs the structural evolution of SnS, Figure 4 contrasts two growth pathways: direct growth on WSe_2_ (Path 2) and buffer‐mediated growth via SnS_2_ (Path 3). Figure 4a–d shows the direct growth pathway (Path 2), where orthorhombic SnS nucleates directly onto the WSe_2_ substrate. Because the SnS layer is immediately subjected to lattice and symmetry mismatch at the interface, compressive strain accumulates within the initial SnS nuclei. As growth proceeds, this interfacial strain is partially relaxed through two distinct mechanisms. As shown in Figure 4a, strain can be accommodated via local lattice distortion within the thermodynamically stable α‐SnS phase. Alternatively, as depicted in Figure 4b, strain relaxation may occur through a structural phase evolution from α‐SnS to the metastable β'‐SnS phase, which adopts a different stacking registry that lowers elastic energy.

These two strain‐relaxation mechanisms are directly observed in cross‐sectional HAADF‐STEM images. Figure 4c presents an α‐SnS layer grown directly on WSe_2_, where pronounced lattice distortion is visible near the interface (highlighted region by white dashed lines). The distorted atomic arrangement indicates variation in Sn–S interatomic distances relative to the ideal α‐SnS structure. Importantly, this distortion is not confined to the immediate interface but propagates vertically into the overgrown SnS layer, suggesting incomplete strain relaxation at the vdW interface. Such vertical strain propagation is consistent with prior reports on van der Waals epitaxy, where lattice mismatch at the initial growth stage significantly influences the structural evolution of the subsequent layers [8, 9]. Additional magnified images and intensity profiles are provided in Figures S6 and S7.

Figure 4d further reveals strain‐driven phase evolution within directly grown SnS. Viewed along the zigzag direction, a clear structural transition is observed across the white dashed boundary: the lower region adjacent to WSe_2_ retains the zigzag stacking characteristic of α‐SnS, whereas the upper region adopts the dumbbell‐like atomic configuration of β′‐SnS. As illustrated by the atomic models (Figure S8), this transition originates from a change in stacking registry, where Sn atoms in β′‐SnS undergo lateral shifts relative to the vertically aligned stacking in α‐SnS. Although α‐SnS is the thermodynamically stable phase under strain‐free conditions, the β′ phase can be stabilized through strain accommodation, indicating that substrate‐induced strain significantly alters phase stability even in van der Waals epitaxy as reported elsewhere [16, 17, 21, 25].

In sharp contrast, Figure 4e,f and Figure S9 illustrate the buffer‐mediated growth strategy (Path 3). By simultaneously ramping T_1_ and T_2_ to initially enter the SnS_2_‐stable region of the phase map, a layered SnS_2_ film nucleates first on WSe_2_, forming a vdW buffer layer prior to SnS growth. As the growth trajectory subsequently crosses into the SnS‐stable regime, orthorhombic SnS nucleates on the pre‐formed SnS_2_ layer, resulting in a vertical SnS/SnS_2_/WSe_2_ heterostructure. The cross‐sectional HAADF‐STEM image in Figure 4f reveals an atomically sharp interface and a distortion‐free α‐SnS lattice throughout the entire overlayer. Unlike the direct‐growth case, no local lattice distortion or phase evolution is observed. These results demonstrate that insertion of an SnS_2_ buffer layer effectively decouples the propagation of substrate‐induced strain to SnS overlayer, preventing both lattice distortion and strain‐driven phase evolution. Consequently, pathway‐controlled growth enables deterministic control over not only phase selection but also interfacial strain accommodation, allowing the realization of structurally pristine vdW heterostructures even in systems with substantial lattice mismatch.

## 3 Conclusion

In this study, we have demonstrated pathway‐controlled vdW epitaxy of tin sulfides using a two‐zone chemical vapor deposition system that allows independent and time‐resolved control of sulfur and tin precursor supply. By modulating the growth pathway within a well‐defined phase map, we selectively synthesized SnS, SnS_2_, and vertically stacked SnS/SnS_2_ heterostructures. Our results reveal that direct growth of SnS on WSe_2_ induces substrate‐mediated strain, leading to local lattice distortion and strain‐driven phase evolution, whereas introducing a pre‐formed SnS_2_ interlayer enables deformation‐free stabilization of the thermodynamically favored α‐SnS phase.

More importantly, this work uncovers that even in van der Waals epitaxy, where lattice matching constraints are often presumed to be negligible, the growth pathway critically governs strain transfer, phase stability, and structural integrity at the heterointerface. By decoupling epitaxial alignment from strain accommodation through pathway engineering, our approach provides a general design principle for stabilizing strain‐sensitive or metastable phases in lattice‐mismatched vdW heterostructures. Furthermore, this pathway‐controlled strategy could be applied to various stoichiometries or symmetry‐mismatched systems, allowing the synthesis of complex vdW heterostructures. Beyond the Sn–S system, pathway‐controlled vdW epitaxy offers a versatile and scalable framework for engineering phase‐, composition‐, and stacking‐controlled chalcogenide heterostructures, with broad implications for functional electronic, optoelectronic, and ferroelectric devices based on low‐dimensional materials.

## 4 Methods

### 4.1 Van der Waals Epitaxial Growth

#### 4.1.1 WSe_2_ Template Growth

WSe_2_ template was synthesized on SiO_2_/Si substrates with a 285 nm SiO_2_ layer by a conventional CVD process conducted in a 2‐inch quartz tube under atmospheric pressure. For the growth of WSe_2_, 200 mg of WO_2.9_ powder (99.99%, Thermo Fisher Scientific) was mixed with 20 mg of KI powder (99.9%, Alfa Aesar) as a growth promoter and placed at the center of the furnace. A SiO_2_/Si substrate was positioned face‐down above the powder. A separate quartz boat containing 100 mg of selenium powder (99.99%, Sigma–Aldrich) was placed upstream in the tube, 20 cm away from the furnace center. The furnace temperature was ramped to 850°C at a rate of 50°C/min, maintained for 10 min, and then naturally cooled to room temperature. Throughout the entire growth process, argon (Ar) and hydrogen (H_2_) gases were continuously supplied at flow rates of 300 and 20 sccm, respectively.

#### 4.1.2 Synthesis of SnS, SnS_2_, and SnS/SnS_2_ Heterostructures

WSe_2_ grown on SiO_2_/Si substrates was used as a van der Waals epitaxy template in a two‐zone CVD system. As the tin precursor, a 10 nm‐thick SnO_2_ film deposited by sputtering on a SiO_2_/Si substrate was employed and placed at the center of Zone 2 on a 3 cm × 3 cm quartz plate. The WSe_2_/SiO_2_/Si target substrate was positioned face‐down above the SnO_2_‐coated source substrate using sapphire spacers as supports. The SnO_2_‐coated source substrate (∼525 µm thick) was cut smaller than the target substrate, and sapphire spacers (∼650 µm thick) were placed at both edges of the source substrate to prevent direct physical contact while maintaining a well‐defined gap between the source and target substrates. In Zone 1, a quartz boat containing 100 mg of sulfur powder (Sigma–Aldrich) was placed at the center of the furnace, with a source‐to‐substrate distance of 32.5 cm.

#### 4.1.3 Growth of SnS_2_ (Path 1)

To synthesize SnS_2_ on WSe_2_, Zone 1 was heated to 160°C at a ramping rate of 16°C/min while Zone 2 was simultaneously heated to 400°C at a ramping rate of 40°C/min and maintained for 5 min.

#### 4.1.4 Growth of SnS (Path 2)

To synthesize SnS on WSe_2_, Zone 2 was first heated at a ramping rate of 40°C/min, while Zone 1 was maintained at room temperature to suppress sulfur evaporation. When the temperature of Zone 2 exceeded 200°C, Zone 1 was subsequently ramped to 120°C at a rate of 20°C/min and maintained for 5 min, while Zone 2 continued to ramp at the same rate to a final temperature of 400°C. Zone 2 was held at 400°C for 5 min to complete the growth.

#### 4.1.5 Synthesis of Vertical SnS/SnS_2_/WSe_2_ Heterostructure (Path 3)

For the synthesis of vertical SnS/SnS_2_ heterostructures, Zone 1 was ramped to 120°C at a rate of 10°C/min and maintained for 5 min, while Zone 2 was simultaneously heated to 400°C at a ramping rate of 40°C/min. Throughout all growth processes, argon (Ar) and hydrogen (H_2_) were continuously supplied at flow rates of 300 and 100 sccm, respectively. At the end of all growth processes (SnS, SnS_2_, SnS/SnS_2_), the furnaces were naturally cooled to room temperature.

### 4.2 Raman and PL Spectroscopy

Raman and photoluminescence (PL) spectra were obtained by using a Raman spectrometer (JASCO, NRS‐4500) with a laser of wavelength 532 nm (diode‐pumped solid‐state laser), spot size of 1 µm. Angle‐resolved polarized Raman spectra were measured by fixing the polarization of the incident laser and rotating the sample by 15° to vary the angle between the incident laser polarization and the crystal axes. The scattered light polarization was analyzed in a parallel‐polarized configuration, and spectra were collected as a function of rotation angle.

### 4.3 X‐Ray Photoelectron Spectroscopy

XPS spectra of Sn and S were obtained using a 10 µm beam spot size with Al‐Kα radiation (ULVAC‐PHI, VersaProbe III).

### 4.4 Atomic Force Microscopy

AFM image was obtained using an NX‐10 (Park Systems). Non‐contact mode was performed considering the status of the samples and the environment.

### 4.5 TEM Imaging

Samples for TEM imaging were prepared using a wet‐transfer method based on poly methyl methacrylate (PMMA). Initially, the samples were spin‐coated with PMMA solution and annealed at 180°C for 2 min. PMMA coated substrates were etched with KOH solution and rinsed with DI water and transferred onto a holey Au TEM grid. After evacuating the TEM grid for 6 h, samples were immersed in acetone for 24 h to remove PMMA. TEM/HAADF‐STEM imaging was performed using Cs‐STEM (Thermo Fisher, Themis Z) operating at 200 kV, and HAADF‐STEM images were acquired with a probe current of 50 pA. A focused ion beam (FIB) system (Thermo Fisher, Helios G4) was used to fabricate cross‐section STEM samples.

## Author Contributions

J.L. and J.K. contributed equally. J.L., J.K., and G.‐H.L. designed the project. J.L and J.K. synthesized samples by CVD and performed Raman measurements. J.L. performed statistical analysis of SnS, and J.K. performed TEM imaging and XPS measurements. J.L., J.K., and G.‐H.L. analyzed the data and wrote the manuscript together.

## Conflicts of Interest

The authors declare no conflicts of interest.

## Supporting information




**Supporting File**: advs76199‐sup‐0001‐SuppMat.docx.

## Data Availability

The data that support the findings of this study are available from the corresponding author upon reasonable request.
