# Supplementary material for: Growth‐Pathway‐Controlled van der Waals Epitaxy of Phase‐Selective Tin Sulfides
Source: Adv Sci (Weinh). 2026 Jun 26:e76199. Online ahead of print. doi: 10.1002/advs.76199 (PMC13336665; doi:10.1002/advs.76199)
Supplement: Supplementary file 1 — Supporting File: advs76199‐sup‐0001‐SuppMat.docx. [file ADVS-9999-e76199-s001.docx]

**Supporting Information**

Growth-pathway-controlled van der Waals epitaxy of phase-selective tin sulfides

Jaehyeok Lee^1, ‡^, Jinwoo Kim^1, ‡^, Gwan-Hyoung Lee^1, *^

^1^ Department of Material Science and Engineering, Seoul National University, Seoul 08826, Republic of Korea


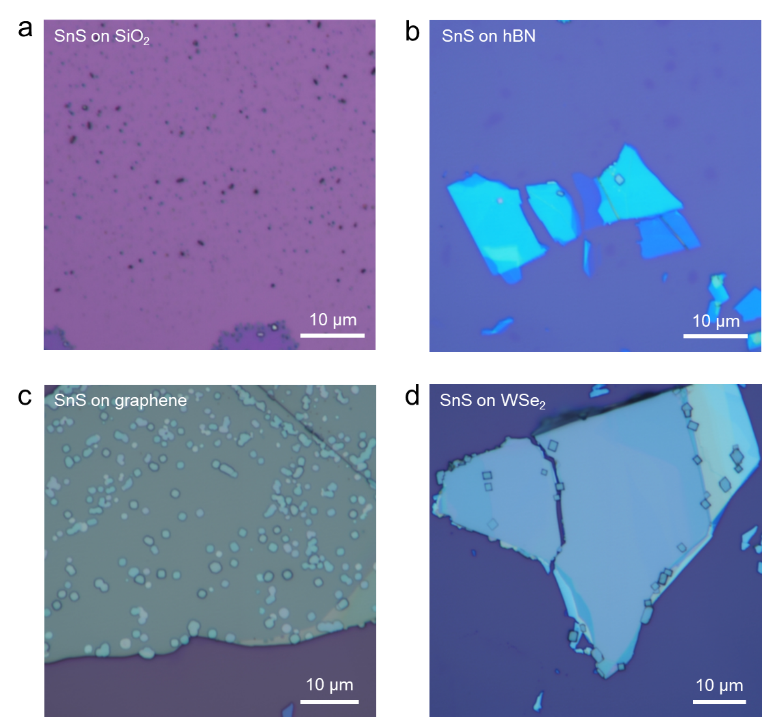

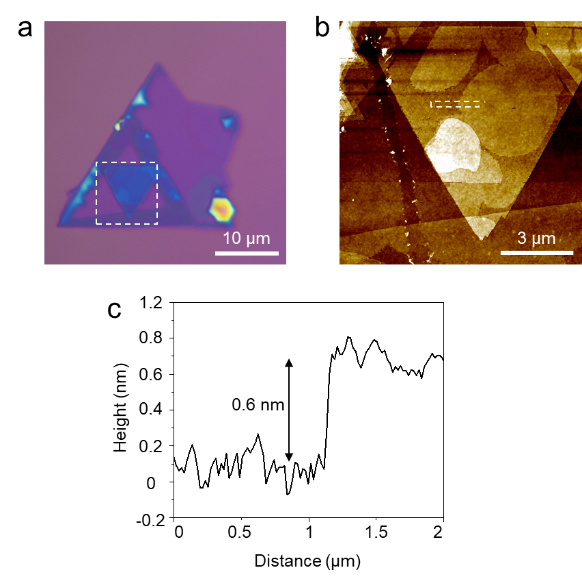

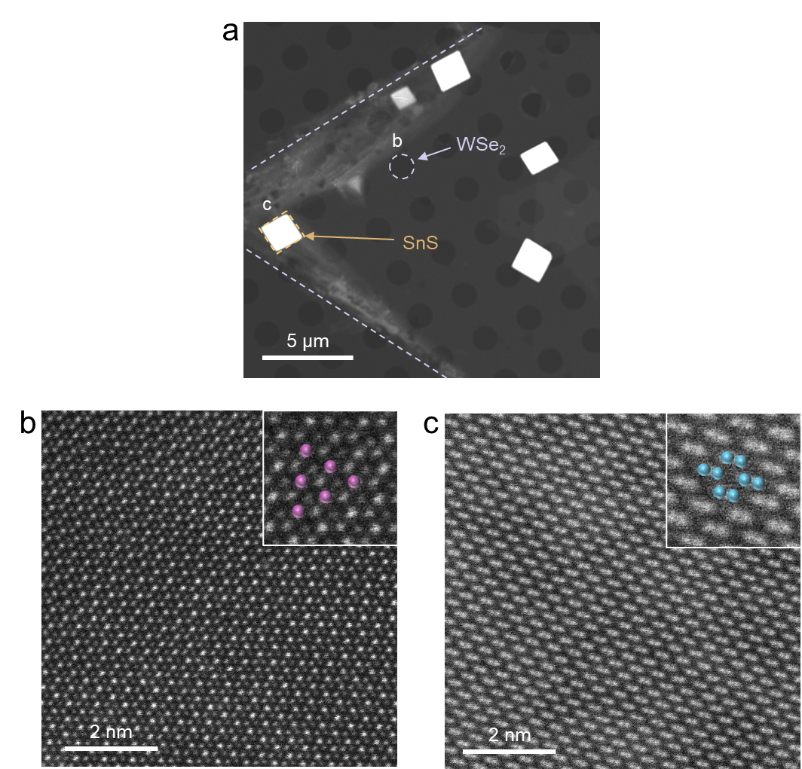


Figure S1. Optical microscopy (OM) image of SnS grown on various substrate. (a-d) Optical microscopy (OM) image of SnS grown on (a) SiO_2_, mechanically exfoliated (b) hBN, (c) graphene, (d) WSe_2_, following the pathway for SnS growth with T_1_ = 100 ℃ and T_2_ = 450 ℃.

Figure S2. Atomic force microscopy (AFM) image of SnS_2_ grown on WSe_2_. (a) Optical microscopy (OM) image of SnS_2_ grown on WSe_2_. (b) Atomic force microscopy (AFM) image of SnS_2_/WSe_2_, indicated with whited dashed box in (a). (c) Height profile of SnS_2_/WSe_2_ of highlighted with white dashed box region from (b), confirming 1L thickness of SnS_2_.

Figure S3. Top view HAADF-STEM image of SnS on WSe_2_. (a) Low-magnification HAADF-STEM images of SnS grown on a WSe_2_ substrate. (b) High-resolution HAADF-STEM image of WSe_2_. The inset shows W atoms. (c) HAADF-STEM image from the region of SnS/WSe_2_. Only SnS is visible in top view HAADF-STEM image due to thickness of SnS.


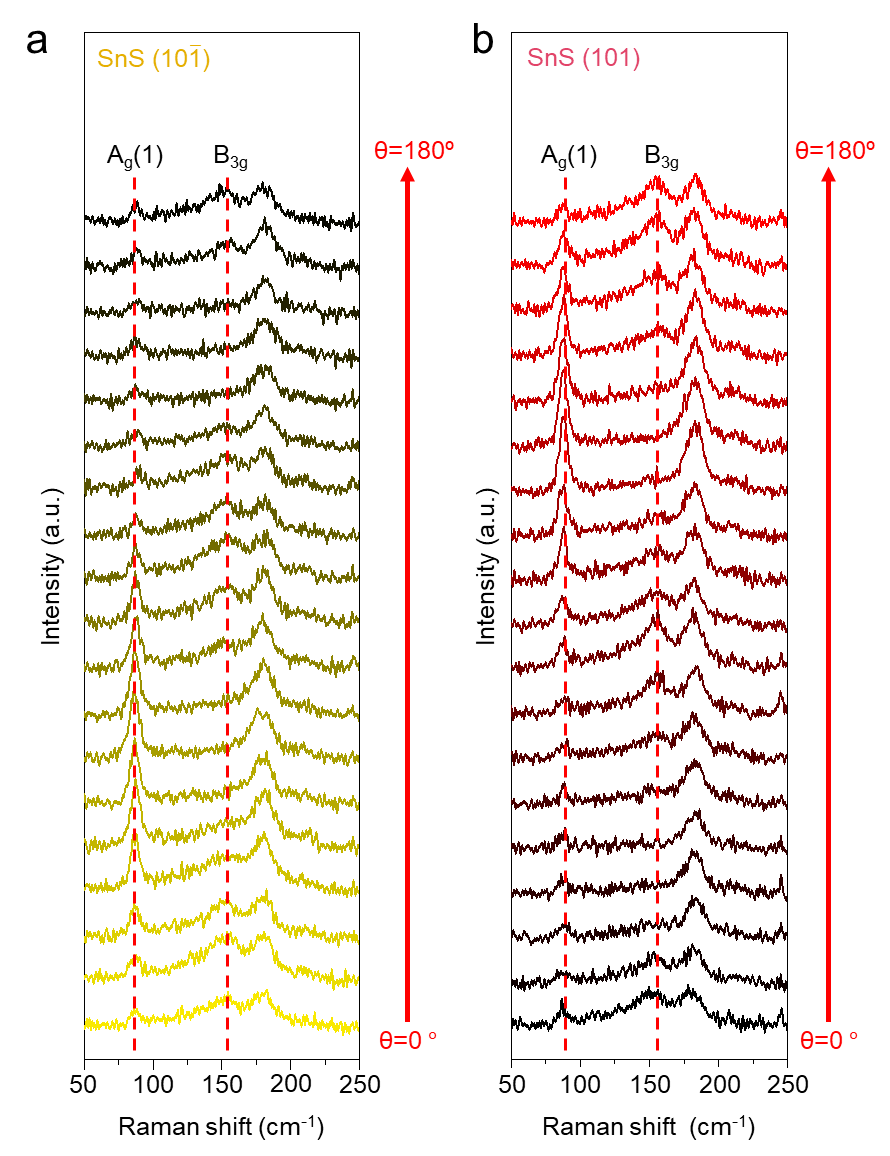


Figure S4. Polar plot of angle-dependent polarized Raman spectra of two representative SnS flakes in Figure 3. Polar plot of angle-dependent polarized Raman spectra of the (a) SnS $(10\overline{1})$, and (b) SnS (101) flake from 0º to 180º, showing the A_g_(1) mode with 2-fold symmetry along the armchair direction.


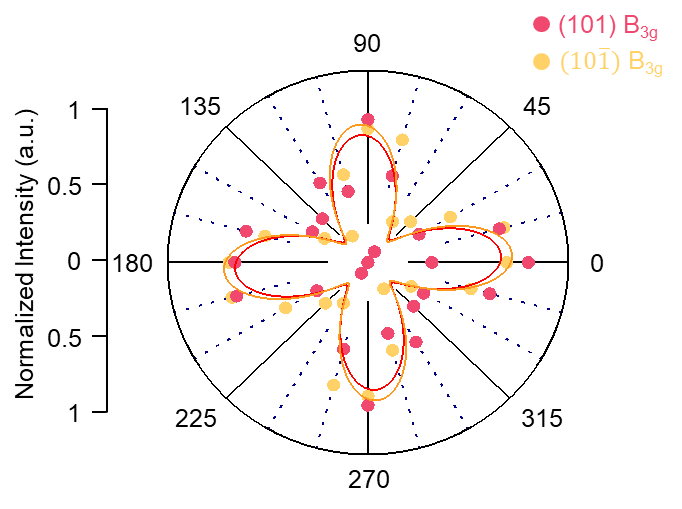


Figure S5. Polarized Raman spectroscopy of SnS/WSe_2_ heterostructure. A Polar plot of the normalized Raman intensities for the B_3g_ vibration mode of the SnS $(10\overline{1})$ and (101) domains, showing 4-fold symmetry.


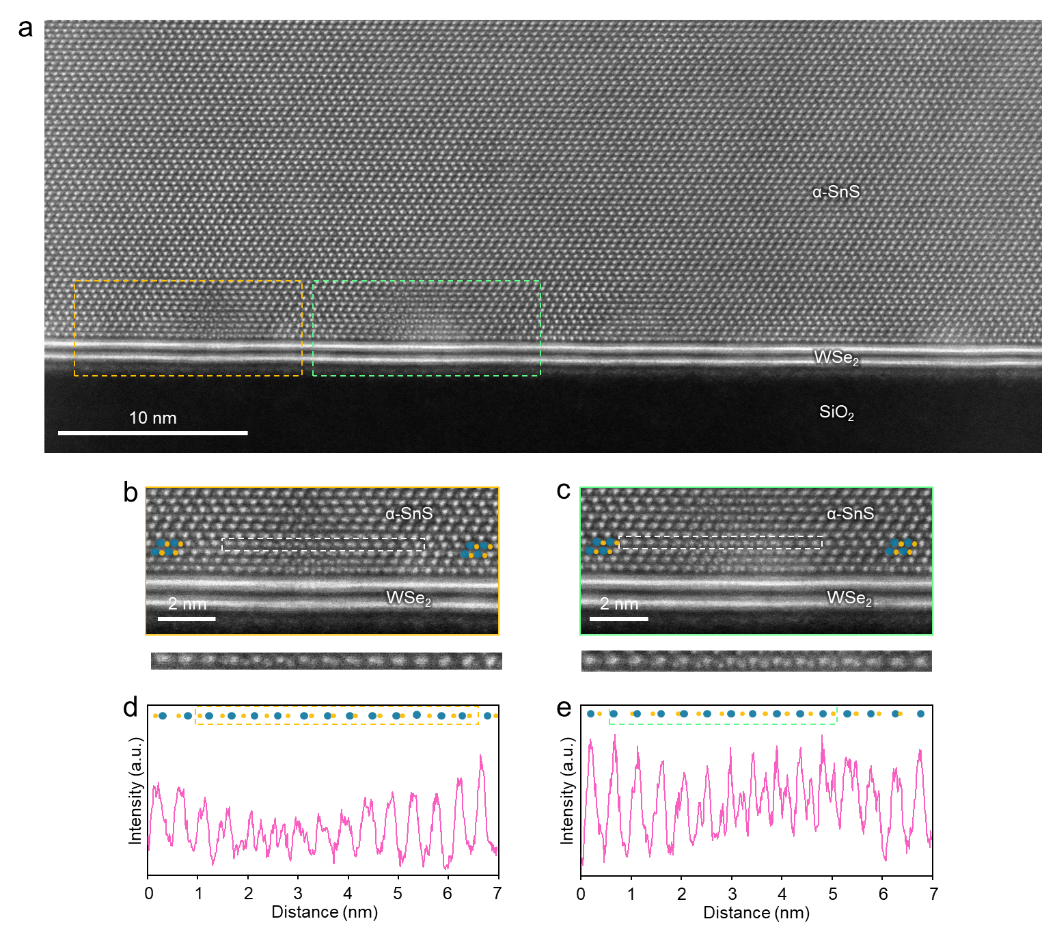


Figure S6. Atomic-scale analysis of interface between SnS and WSe_2_. (a) Low-magnification cross-sectional HAADF-STEM image of the SnS layer grown WSe_2_, showing localized structural distortions at the heterointerface. (b, c) Magnified HAADF-STEM images corresponding to the (b) orange and (c) green dashed boxes in (a), highlighting the interfacial distortions. Note that the SnS domain orientation remains consistent despite the presence of local distortions. (d, e) Intensity line profiles obtained from the white dashed regions in (b) and (c), respectively, with overlaid atomic models. The profiles indicate variations in Sn–S interatomic distances, reflecting the local strain distribution within the distorted regions. Despite these localized distortions, the domain orientation of α-SnS remains constant throughout the crystals, reflecting a single domain structure of α-SnS.


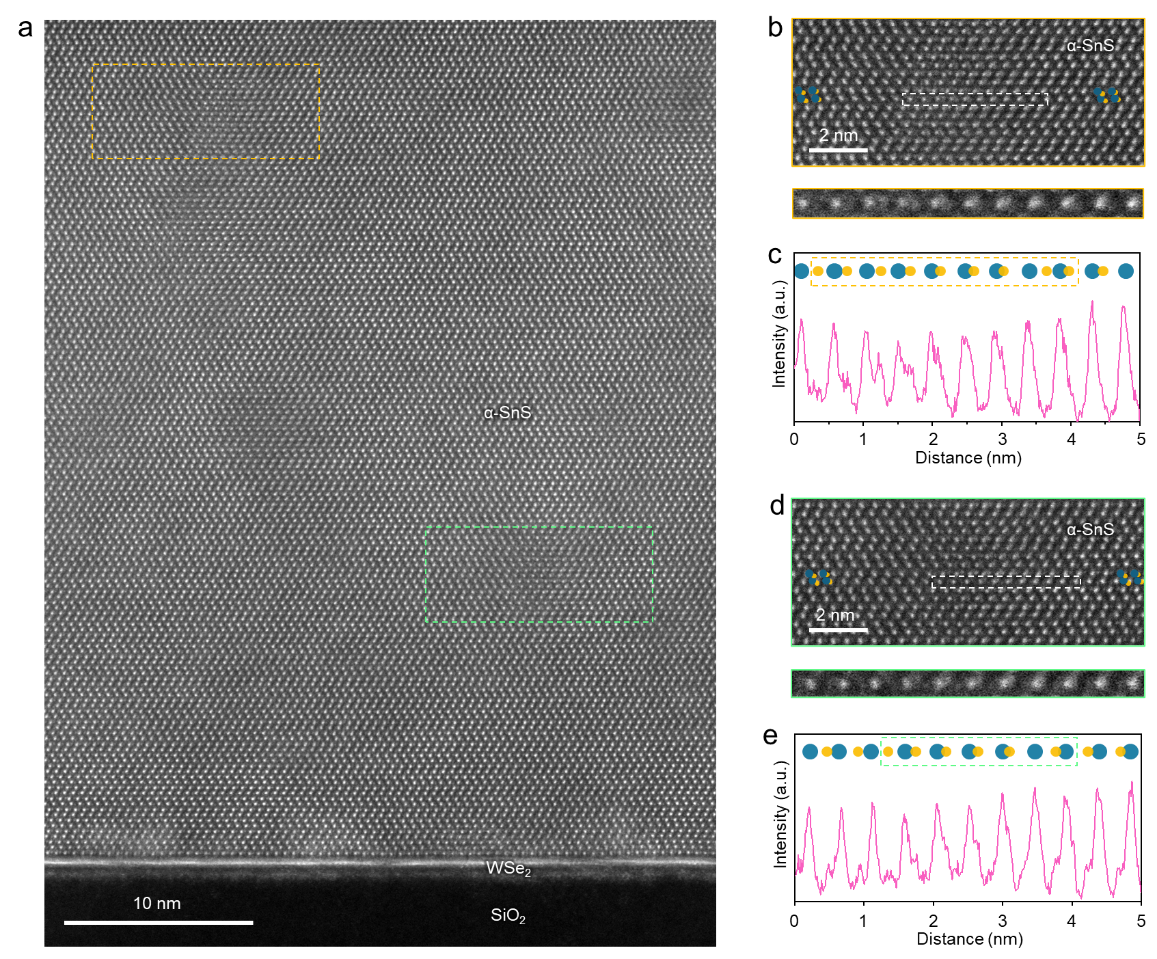


Figure S7. Atomic-scale analysis of distortion in bulk SnS. (a) Low-magnification cross-sectional HAADF-STEM image of the SnS layer grown on WSe_2_, showing structural distortions at bulk region. (b, d) Magnified HAADF-STEM images corresponding to the (b) orange and (d) green dashed boxes in (a), highlighting the structural distortions. Note that the SnS domain orientation remains consistent despite the presence of local distortions. (c, e) Intensity line profiles obtained from the white dashed regions in (b) and (d), respectively, with overlaid atomic models. The profiles indicate variations in Sn–S interatomic distances, reflecting the local strain distribution within the distorted regions. Despite these localized distortions, the domain orientation of α-SnS remains constant throughout the crystals, reflecting a single domain structure of α-SnS.


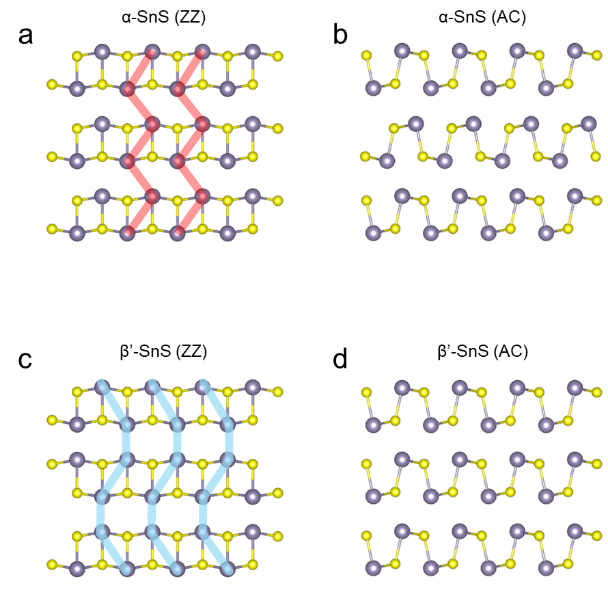


Figure S8. Atomic model of α-SnS and β'-SnS phases. (a-d) Crystal structures viewed along the zigzag (ZZ) and armchair (AC) directions of SnS with two different phases. (a) α-SnS along ZZ direction, (b) α-SnS along AC direction, (c) β'-SnS along ZZ direction, (d) β'-SnS along AC direction. The colored lines in (a) and (c) trace the Sn atoms configurations along the ZZ direction, illustrating the distinct atomic arrangement across the phase boundary observed in STEM images of Figure 4d.


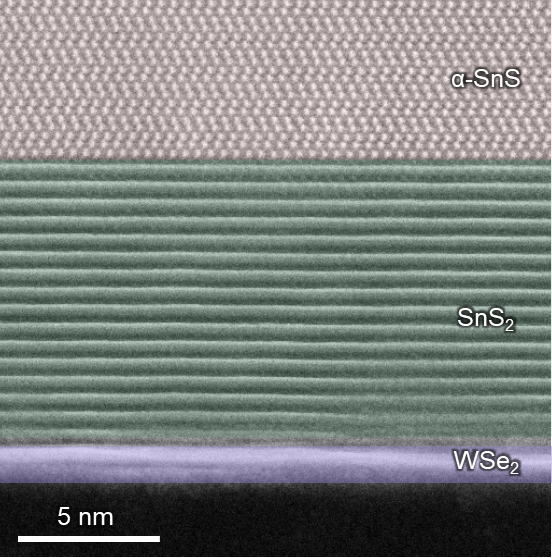


Figure S9. Cross-sectional HAADF-STEM image of the SnS/SnS_2_/WSe_2_ heterostructure corresponding to Path 3, obtained from a different region than that shown in Figure 4f. The vertically stacked SnS/SnS_2_/WSe_2_ and their interfaces are clearly resolved in the HAADF-STEM image. No obvious structural distortion or extended interfacial mixing is observed across the heterointerfaces.
